# A Sperm–Plasma β-*N*-Acetyl-D-Hexosaminidase Interacting with a Chitinolytic β-*N*-Acetyl-D-Hexosaminidase in Insect Molting Fluid

**DOI:** 10.1371/journal.pone.0071738

**Published:** 2013-08-12

**Authors:** Mingbo Qu, Tian Liu, Peng Chen, Qing Yang

**Affiliations:** School of Life Science and Biotechnology, Dalian University of Technology, Dalian, China; Goethe University Frankfurt, Germany

## Abstract

Insects require molting fluids to shed the old cuticle during molting. β-*N*-acetyl-D-hexosaminidase, known as Hex1, together with various chitinases, is responsible for degrading the chitin component of the old cuticle. This study showed that another β-*N*-acetyl-D-hexosaminidase, termed OfHex3, interacted with Hex1 and functioned in the molting fluid, although the homolog of OfHex3 was known as a sperm–plasma enzyme functioning in egg–sperm recognition. OfHex3 is an enzyme cloned from the insect Asian corn borer, *Ostrinia furnacalis*, which is one of the most destructive pests of maize. The enzymatic activity analysis indicated that OfHex3 was able to degrade chitooligosaccharides, but at a lower rate than that of OfHex1. Because OfHex3 did not have substrate inhibition, we deduced that the presence of OfHex3 might help OfHex1 relieve substrate inhibition during chitin degradation during molting. The expression patterns of OfHex3 during *O. furnacalis* development were studied by real-time PCR as well as western blot. The results showed that both gene transcription and protein translation levels of OfHex3 were up-regulated during larval–larval molting. The tissue-specific expression pattern analysis indicated that OfHex3 was mostly localized in the fat body and testis. All these data further supported that Hex3 was involved in molting as well as in fertilization. This study may help to understand the complexity of cuticle degradation during insect molting, and may provide a possible target for pest control.

## Introduction

Molting is an important process in insect growth and development [1]. To molt, an insect synthesizes and secrets molting fluids into a lumen located between the old and new cuticle [2]. Because chitin is the primary component of insect cuticle, one of the main functions of molting fluid is to hydrolyze this highly polymerized saccharide. The chitinolytic activity of molting fluids was first reported in the exuvia fluid of *Bombyx mori* (Lepidoptera) [2], [3], then two kinds of chitinolytic enzymes, previously termed endo-chitinase and exo-chitinase but now named chitinase (EC3.2.1.14) and β-*N*-acetyl-D-hexosaminidase (Hex; EC 3.2.1.52), respectively, were isolated and characterized. Chitinases degrade chitin into oligosaccharides, and Hexes degrade oligosaccharides to *N*-acetyl-β-D-glucosamine (GlcNAc) [4].

Insect genomes contain as many as seven genes encoding Hex that function during different life processes. These genes fall into four phylogenetic classes [5]. The class I Hexes (Hex1s) possess chitinolytic activities and have been found in several insects [6], [7], [8]. It occurs in dimeric and glycosylated form [6]. Studies of the enzymatic properties of Hex1 from both *Manduca sexta* (Lepidoptera) and *B. mori* suggested that it could degrade (GlcNAc)_6_ to GlcNAc [9] and was more active toward the synthesized substrate *p*NP-β-GlcNAc (*p*-nitrophenyl-*N*-acetyl-β-D-glucosaminide) than toward *p*NP-β-(GlcNAc)_2_ (*p*-nitrophenyl -β-D-chitobiose) [6]. The first crystal structure of an insect Hex1, OfHex1 from *Ostrinia furnacalis* (Lepidoptera), provided an elegant structural explanation for why chitinolytic Hex1s possess highly catalytic activity towards chitooligosaccharide substrates [10]. The expression pattern of *HEX1* was also studied. In *M. sexta*, *MsHEX1* was mainly expressed in the epidermis on the sixth and seventh days of the fifth instar [11]. In *Tribolium castaneum* (Coleoptera), RNA interference against *TcHEX1* interrupted larval–larval, larval–pupal, and pupal–adult development [5]. In *Choristoneura fumiferana* (Lepidoptera), immunohistochemistry staining indicated that CfHex1 was localized in molting fluid, epidermis and trachea [12]. Taken together, these data demonstrated that Hex1 is an enzyme involved in chitin degradation.

Here, we cloned another Hex, named *OfHEX3*, from the Asian corn borer, *O. furnacalis*, an important lepidopteran pest of maize and cotton. OfHex3 belongs to class III Hexes. Although previously reported class III enzymes were involved in sperm–egg interaction [13], [14], [15], [16], this study found that OfHex3 occurred with OfHex1 in molting fluids. Enzymatic activity and expression pattern both suggested that besides functioning in fertilization, OfHex3 worked with OfHex1 in cuticle degradation.

## Materials and Methods

### Molecular Cloning and Sequence Analysis of *OfHEX3*

Total RNA was isolated from fifth-instar larvae of *O. furnacalis* using RNAiso Reagent (TaKaRa, Dalian, China). cDNA was synthesized using 3′-Full RACE Core Set Ver.2.0 (TaKaRa) from 2 µg total RNA using random hexamer primers and oligo-(dT) as reverse transcript primers. Gene-specific primers were designed based on conserved sequences within insect Hexes (Table S1) and used for PCRs (Figure S1). The 5′- and 3′- ends were obtained using 3′-Full RACE Core Set Ver.2.0 and 5′-Full RACE Kit (TaKaRa), respectively.

The signal peptide of OfHex3 was predicted by Signal P 4.0 program [17]. Structure-based multiple sequence alignments of OfHex3 and other insect Hexes were performed with PROMALS3D [18] using the crystal structure of OfHex1 (PDB code: 3NSM) as structure input. Sequence alignment was performed by using the software ESpript 2.2 [19]. Phylogenetic trees of insect Hexes were constructed with MEGA 5 [20] using the neighbor-joining method with a 5,000 bootstrap replicates.

The structure model of the catalytic domain of OfHex3 was generated by Modeller 9.10 [21] using the catalytic domain of OfHex1 as template [10]. The best of 20 generated models was selected and refined using the loop refinement module in Modeller 9.10 according to the reports by PROCHECK [22] and Verify_3D [23]. The structure figure was prepared using PyMOL.

### Recombinant Expression and Purification of OfHex3

*OfHEX3* cDNA lacking the first 54 nucleotides encoding the signal peptide was cloned into pPIC9 vector (Invitrogen, Carlsbad, CA, USA) between the *EcoR* I and *Not* I sites with α-factor at the N-terminus and 6×His-tag at the C-terminus in frame. The generated plasmid, named pPIC9-OfHex3, was linearized using *Pme* I and transformed into *Pichia pastoris* strain GS115 by electroporation. Positive clones were selected as previously described [24].

The selected recombinant *P. pastoris* was precultured in BMGY medium and then transferred to BMMY medium for inducible expression by 1% methanol according to the manufacture's instruction. After 144 h of induction, the recombinant protein was obtained first by 75% saturation of ammonium sulfate precipitation and then by affinity chromatography on IMAC Sepharose High Performance column (5 mL, GE Healthcare) [24]. The purity of recombinant OfHex3 was analyzed by 10% (w/v) SDS–PAGE. For determination of deglycosylation, 20 µg of the purified OfHex3 were denatured and reacted with either 2 µL glycopeptidase F (TaKaRa) or 2 µL water according to the manufacturer's instruction, and then analyzed by 10% SDS–PAGE.

### Enzymatic Assay

For the substrates *p*NP-β-GlcNAc and *p*NP-β-GalNAc (*p*-nitrophenyl-*N*-acetyl-β-D-galactosaminide) (Sigma-Aldrich, St. Louis, MO, USA), the reaction mixture contained substrate (0.05–5 mM) and 2 µL enzyme in 60 µL of Britton–Robinson’s wide range buffer (pH 2–12). After incubation at 25°C for 30 min, the reaction was stopped by adding 60 µL of 0.5 M Na_2_CO_3_ and absorbance was measured at 405 nm using a Sunrise microplate reader (Tecan, Männedorf, Switzerland). The reaction velocity was quantified by comparing the absorbance of the product *p*NP (*p*-nitrophenol) with a standard curve of *p*NP with known concentrations.

For the substrates (GlcNAc)_n_ (n = 2–4), GlcNAcβ1,2Man, and GlcNAcβ1,3GalNAc (Toronto Research Chemicals, Toronto, Canada), the enzymatic reaction mixture consisted of 30 µL substrate (0.1–1 mM) in 5 mM sodium phosphate buffer, 25 µL of 5 mM sodium phosphate buffer (pH 6.0), and 5 µL of enzyme. The reaction mixtures were incubated at 25°C for an appropriate time, and then the reaction was immediately stopped by incubation on ice. The hydrolysis products were analyzed by HPLC using a TSKgel Amide-80 column [25]. The hydrolytic products were quantified by converting the peak area to concentration values according to a standard curve of GlcNAc made with known concentrations. Enzymatic reactions were terminated before 15% of substrate was consumed. The *K*_m_ and *k*_cat_ values were also calculated by linear regression of the data using Lineweaver–Burk plots.

For the pyridylaminated (PA) substrates GnGn [GlcNAcβ1,2Manα1,6(GlcNAcβ1,2Manα1,3)Manβ1,4GlcNAcβ1,4GlcNAc], GA2 [GalNAcβ1,4Galβ1,4Glc] and Gb4 [GalNAcβ1,3Galα1,4Galβ1,4Glc], the enzymatic reaction mixture consisted of 25 µL of 10 µM substrate in 5 mM sodium phosphate buffer, 20 µL of 5 mM sodium phosphate buffer (pH 6.0), and 5 µL of enzyme. The reaction mixtures were incubated at 25°C for 24 h and the hydrolysis products were analyzed by HPLC as previously described [25].

### Expression Pattern of *OfHEX3* During *O. furnacalis* Development

To evaluate the temporal expression pattern of *OfHEX3*, insects ranging from the third-instar day-2 to pupa day-2 were sampled. For tissue–specific expression, samples of the integument, midgut, fat body, trachea, Malpighian tubule, silk gland, and testis of the fifth-instar day-3 larvae were isolated.

Gene–specific primers for real-time PCR were designed according to the most unique sequence of *OfHEX3* (Table S2). The housekeeping gene *OfRpS3* (GenBank ID: EU275206) was chosen as the endogenous control [26]. The standard curves for each gene were generated by serial (5×) dilutions of PCR products. RNAs were isolated from the samples collected and cDNAs were synthesized. Real-time PCR was performed and the expression levels of *OfHEX3* were calculated as described previously [27].

A unique peptide (“KDPTPIVYEPSWVFKC”) of OfHex3 was synthesized and used as an antigen to produce an antibody. This peptide was highly conserved among lepidopteran Hex3s. The OfHex3 antiserum was further purified by affinity purification using peptide-conjugated agarose gel. Proteins were extracted from the samples and separated by 10% SDS-PAGE. Western blotting was performed as described previously [25]. The antibodies used for western blot were 1∶400 dilution for OfHex3 and 1∶2000 dilution for tubulin.

To determine the specificity of Hex3 antibody for *B. mori* Hex3 (BmHex3), the gene encoding *BmHex3* (GenBank ID: NM_001085364) was cloned into the vector pET22b and expressed in *E. coli*. After induction by 1 mM of IPTG for 4 h, cells were collected. Western blot analysis was applied to determine if the antibody for Hex3 could recognize BmHex3. The effect of tissue–specific expression of Hex3 in *B. mori* on protein level was detected using the Hex3 specific antibody. Samples from different tissues of day-5 fifth-instar larvae were collected, including the integument, midgut, fat body, trachea, Malpighian tubule, silk gland, ovary, and testis. Proteins were extracted and western blotting was performed as described above.

### His-tag Pull-down Assay of OfHex1

Molting fluid was collected from pharate pupae of *O. furnacalis* and *B. mori* (unpublished data, Mingbo Qu). Hex3-specific antibody was used to detect protein in the molting fluid. A His-tag pull-down assay was used to detect the interaction between Hex1 and Hex3. The purified OfHex1-His protein was prepared as previously described [24]. In the experimental group, OfHex1-His protein dissolved in binding buffer (20 mM sodium phosphate, 0.5 M NaCl, pH 7.4) was incubated with molting fluid from *B. mori*. One negative control was purified OfHex1-His incubated with phosphate buffered saline (PBS). Another negative control was PBS incubated with molting fluid, so as to exclude the possibility of any proteins bind to the NTA beads. After incubation for 4 h, the samples were loaded on to Ni-NTA beads (GE Healthcare) and incubated for 1 h. After five washes with binding buffer, the beads were washed five times with binding buffer plus 75 mM imidazole. Finally, the protein was eluted with binding buffer containing 250 mM imidazole and separated on 10% SDS-PAGE. Hex3 antibody was used to detect Hex3 in each sample as described above.

### Subcellular Localization of OfHex3 in Insect Cells

OfHex3 was expressed in the insect cell line Sf9 using insect expression vector pIB-V5/His (Invitrogen). Full-length OfHex3 was inserted in pIB-V5/His with a C-terminal V5 tag and 6×His-tag in frame. Following the same strategy as with full-length OfHex3, several N-terminal truncations (Δ19–55, Δ19–104, and Δ19–155) of OfHex3 were also constructed to investigate whether the subcellular localization was influenced by its N-terminal sequence. All the recombinant expression vectors were transfected into Sf9 using Cellfectin II (Invitrogen) according to the manufacturer's instructions. The subcellular localization of the recombinant proteins were determined by immunofluorescence as described previously [28]. Mouse anti-V5 antibody (Invitrogen) was used to detect the recombinant protein expressed and Cy3 conjugated goat anti-mouse antibody was used for visualization.

## Results

### Sequence Analysis of OfHex3

The cDNA of *OfHEX3* was cloned from total RNA of fifth-instar larvae of *O. furnacalis* by RT-PCR and RACEs. Sequence analysis indicated that *OfHEX3* contains an open reading frame of 1827 nucleotides encoding 609 amino acid residues. OfHex3 was predicted by Signal P 4.0 to be a secreted protein with a signal peptide (residues 1–18) [17].

**Figure 1:**
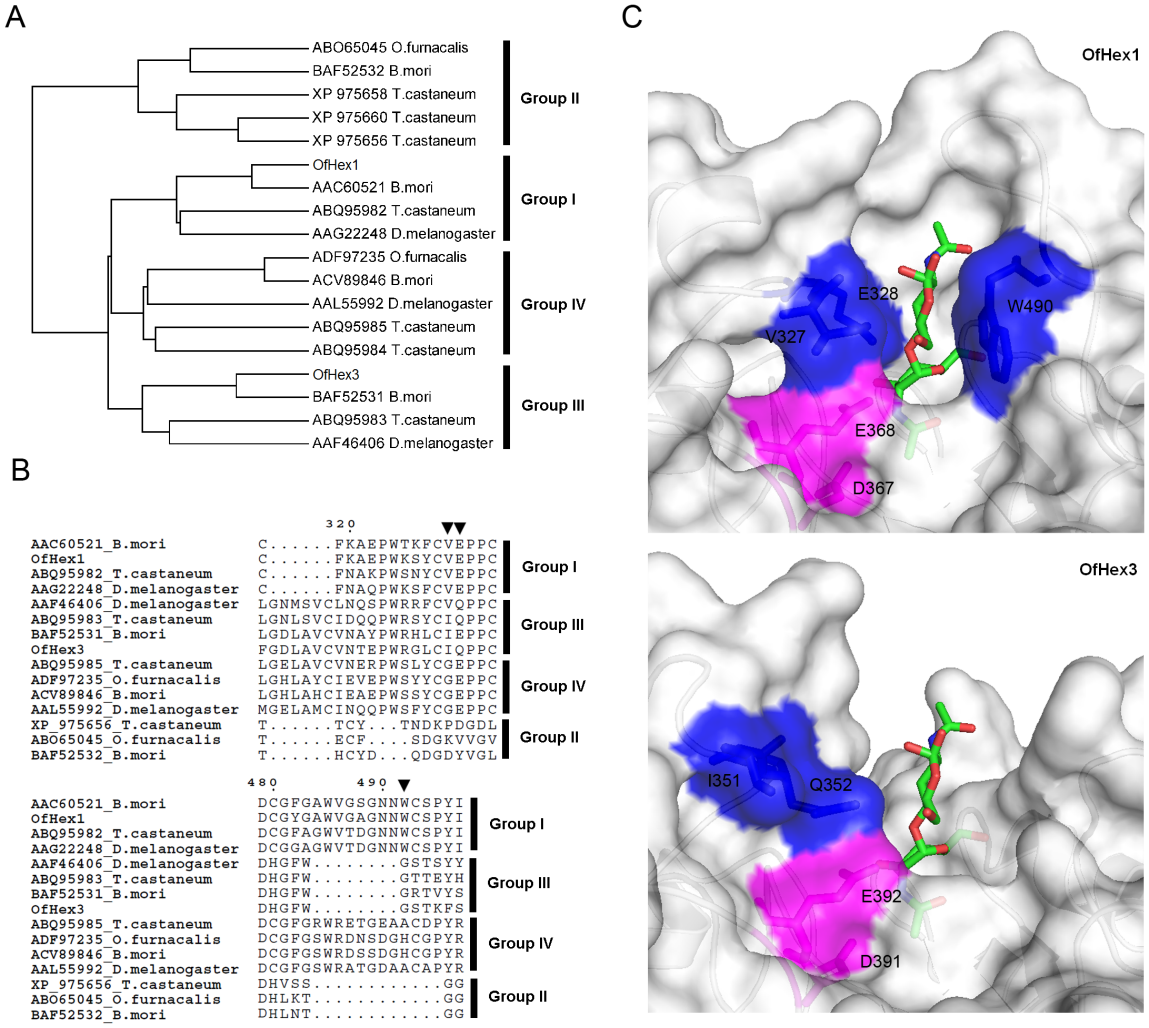
Sequence analysis of OfHex3. (**A**) Phylogenetic relationships of OfHex3 with other insect β-*N*-acetyl-D-hexosaminidases. Phylogenetic tree was constructed with MEGA 5 using the neighbor-joining method with a bootstrap evaluation of 5,000 replications. (**B**) Structure-based multiple sequence alignments of OfHex3 and other insect β-*N*-acetyl-D-hexosaminidases. The important residues for binding +1 sugar in the substrate are labeled with black triangles. (**C**) Comparison of active pocket structures of OfHex1 and OfHex3. The catalytic residues are labeled in pink while the important residues for binding +1 sugar in the substrate are labeled in blue.

The phylogenetic analysis of insect glycoside hydrolase family 20 (GH20) Hexes from four insect species, including *B. mori*, *O. furnacalis*, *Drosophila melanogaster* (Diptera) and *T. castaneum,* indicated these Hexes fell into four groups (Fig. 1A). Group I included chitinolytic Hexes, e.g. OfHex1 (GenBank ID: ABI81756) [29]. Group II comprised Hexes with a broad substrate spectrum, e.g. OfHex2 (GenBank ID: ABO65045) [25]. Group IV consisted of N-glycan modifying Hexes, e.g. DmFDL (GenBank ID: AAL55992) [30]. OfHex3 belonged to Group III, which also included BmHex3 (GenBank ID: BAF52531) [31], DmHexo2 (GenBank ID: AAF46406) [32] and TcNag2 (GenBank ID: ABQ95983) [5].

The structure-based multiple sequence alignment showed that OfHex3 contained all the critical residues that are important for the activity of GH20 Hexes, including Asp^267^, His^321^, Asp^391^ and Glu^392^ for catalysis, Arg^238^, Tyr^503^, Asp^505^ and Glu^541^ for substrate binding and Trp^452^, Trp^476^ and Trp^539^ for substrate binding via stacking interactions (Fig. S2). The most obvious differences among the four groups of insect Hexes were the lengths and amino acid compositions of two loops, L_314–335_ and L_478–496_, located at the entrance of the active pocket of OfHex1 (Fig. 1B). Group III and Group IV Hexes possessed the longest L_314–335_. The residues Val and Glu (black triangles in Fig. 1B), which interacted with the +1 sugar of the substrate in Group I enzymes, were changed to Ile and Gln in the Group III enzymes (Ile^351^ and Gln^352^ in OfHex3), respectively.

The structure model of the catalytic domain of OfHex3 was built using the catalytic domain of OfHex1 (PDB ID: 3NSN) as a template and was validated using PROCHECK (89.8% of the residues were in the most favored regions) and Verify_3D (93.4% of the residues had an average three- to one-dimensional score >0.2). The coordinates of (GlcNAc)_2_ were built based on the structure of the (GlcNAc)_2_ complexed chitobiase from *Serratia marcescens* (PDB ID: 1QBB) [33]. The structures of the active pockets of OfHex1 and OfHex3 were then compared (Fig. 1C). Although catalytic residues (Asp^367^ and Glu^368^ in OfHex1, Asp^391^ and Glu^392^ in OfHex3) were conserved, the key residues comprising the +1 subsites were very different. OfHex3 did not have the aromatic residue (Trp^490^ in OfHex1) to stack with the +1 sugar of the substrate. Unlike in OfHex1, the residues Ile^351^ and Gln^352^ in OfHex3 were set in a position outward of the active pocket such that they could not interact with the +1 sugar of the substrate.

### Recombinant Expression, Purification, and Characterization of OfHex3

The gene encoding mature OfHex3 was successfully expressed in the yeast strain *P. pastoris* GS115 and the recombinant OfHex3 was purified from the culture supernatant with a yield of about 1 mg/L. The purified OfHex3 was resolved as a single band with a molecular weight of 75 kDa by SDS-PAGE analysis and was verified by western blotting using anti-His-tag antibody (Fig. S3). The molecular weight of the recombinant OfHex3 was reduced by 15 kDa after glycopeptidase F treatment (Fig. S3).

**Figure 2:**
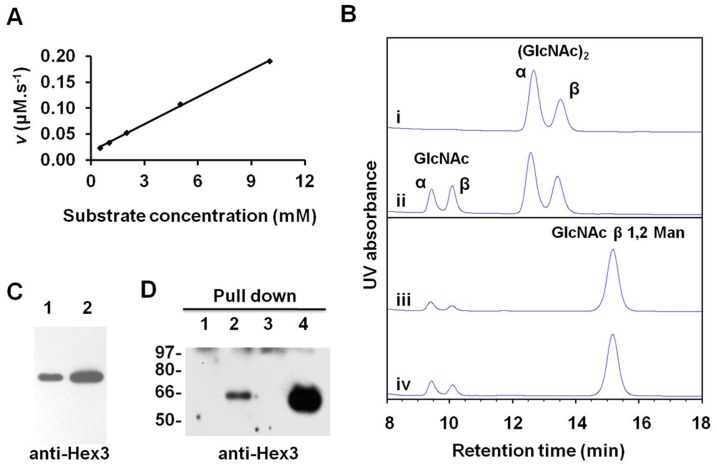
Characterization of recombinant OfHex3. (**A**) No substrate inhibition of OfHex3 using physiological substrate (GlcNAc)_2_. (**B**) HPLC analysis of the enzymatic activities of OfHex3 for (GlcNAc)_2_ (i, ii) and GlcNAcβ1,2Man (iii, iv). The reactions were stopped immediately (i, iii) or after 1 hour (ii, iv) of incubation. (**C**) OfHex3 was detected in molting fluid. Lane 1: molting fluid from *Ostrinia furnacalis*; Lane 2: molting fluid from *Bombyx mori*. (**D**) Pull-down assay with recombinant OfHex1-His protein. Lane 1: purified OfHex1-His incubated with PBS buffer as a negative control; Lane 2: purified OfHex1-His protein incubated with molting fluid from *B. mori*; Lane 3: another negative control that molting fluid from *B. mori* incubated with PBS to exclude the possibility of unspecific binding to the NTA beads; Lane 4: The input of molting fluid from *B. mori* for the pull-down assay. Hex3 was detected by western blot using Hex3 specific antibody.

The kinetic parameters of OfHex3 for *p*NP-β-GlcNAc, *p*NP-β-GalNAc, and (GlcNAc)_2_ were determined. As indicated by *k*_cat_/*K*_m_ values, OfHex3 hydrolyzed the putative physiological substrate, (GlcNAc)_2_, 1.4 times faster than it did *p*NP-β-GlcNAc (Table 1). Moreover, OfHex3 hydrolyzed *p*NP-β-GlcNAc at an 8.1-fold higher efficiency than it did *p*NP-β-GalNAc (Table 1). No substrate inhibition was observed using physiological substrate (GlcNAc)_2_ even with a concentration as high as 10 mM (Fig. 2A).

**Table 1:** Kinetic parameters of OfHex3 for different substrates.

Substrate	*K*_m_ (mM)	*k*_cat_ (s^−1^)	*k*_cat_/*K*_m_ (s^−1^)
*p*NP-β-GlcNAc	1.627	27.74	17.05
*p*NP-β-GalNAc	2.673	5.632	2.107
(GlcNAc)_2_	0.879	21.07	23.97

The glycosidic bond preference of OfHex3 was tested using the substrates GlcNAcβ1,4GlcNAc, GlcNAcβ1,3GalNAc and GlcNAcβ1,2Man. OfHex3 could hydrolyze GlcNAcβ1,4GlcNAc at a rate of 20.6 µM min^−1^, which was 10-fold higher than that of GlcNAcβ1,2Man (Fig. 2B), but it could not hydrolyze GlcNAcβ1,3GalNAc (data not shown).

To determine whether OfHex3 could hydrolyze GlcNAc-containing glycans from glycoproteins and glycolipids, several commercial available PA substrates including GnGn-PA(GlcNAcβ1,2Manα1,6(GlcNAcβ1,2Manα1,3)Manβ1,4GlcNAcβ1,4GlcNAc-PA), GA2-PA (GalNAcβ1,4Galβ1,4Glc-PA) and Gb4-PA (GalNAcβ1,3Galα1,4Galβ1,4Glc-PA) were tested. The results indicated that there were no hydrolytic products after 24 h co-incubation of OfHex3 with these substrates (data not shown).

### OfHex3 Occurred with OfHex1 in Molting Fluids

Since OfHex3 exhibited the activity of hydrolyzing chitooligosaccharides, we tested the molting fluid to see if it contained OfHex3. The molting fluid was collected during larval–pupal molting of *O. furnacalis* and the specific antibody against OfHex3 was applied for western blot analysis. The results showed that OfHex3 was present in the molting fluid (Fig. 2C, lane 1). As Hex1 also occurs in molting fluid, the interaction between Hex3 and Hex1 in molting fluid was tested by pull-down assay.

Here the molting fluid from *B. mori* (BmMF) was used for the pull-down assay instead. *Bombyx mori* is a close relative to *O. furnacalis*, and BmHex3 shares 67% identity in amino acid sequence with OfHex3. The benefit of using *B. mori* is that they are much bigger than *O. furnacalis*, making it easier to collect enough molting fluids. And the specificity of Hex3 antibody towards BmHex3 was confirmed by western blot (Fig. S4). The OfHex3 antibody could also recognize BmHex3 in the molting fluid from *B. mori* (Fig. 2C, lane 2). The results suggested that BmHex3 could be pulled down by OfHex1 with high specificity compared with the control with no bait protein or molting fluid (Fig. 2D, lanes 1–4).

### Expression Pattern of OfHex3 during *O. furnacalis* Development

**Figure 3:**
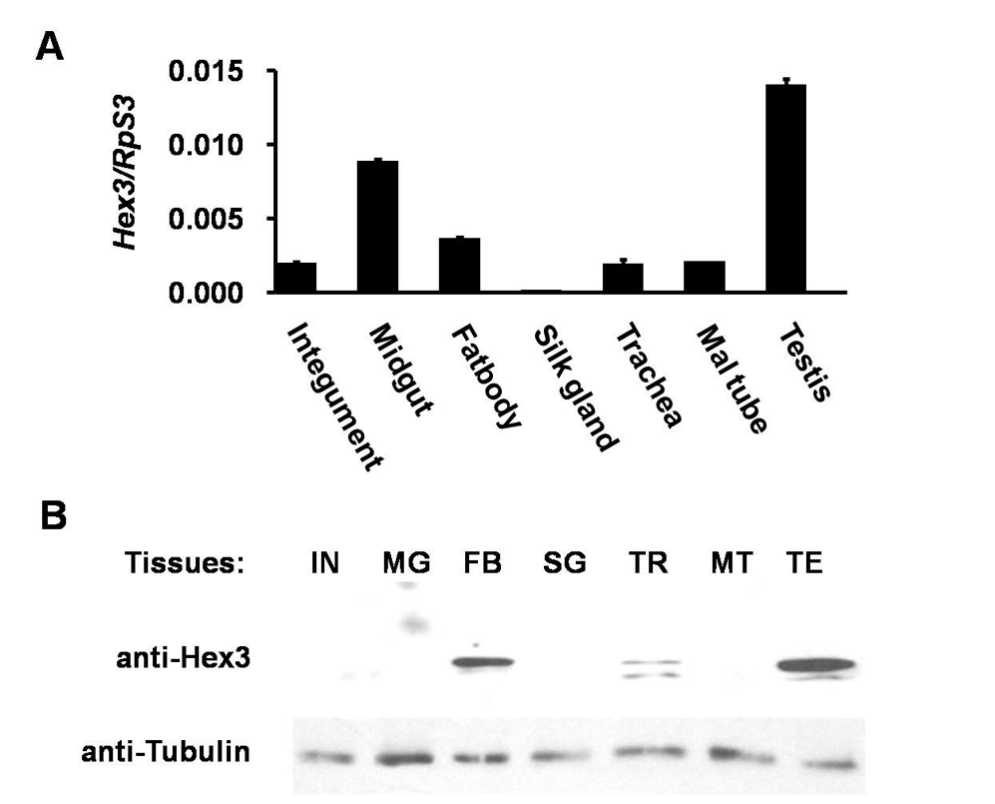
Tissue-specific expression of OfHex3. (**A**) Gene expression levels of *OfHEX3* in different tissues of *Ostrinia furnacalis*. Numbers indicate relative gene expression levels compared with the housekeeping gene *OfRpS3*. (**B**) Protein expression profile of OfHex3 in different tissues of *O. furnacalis*. IN: integument, FB: fat body, MG: midgut, SG: silk gland, MT: Malpighian tubule, TR: trachea, TE: testis. Tubulin was chosen as a loading control.

The expression pattern of OfHex3 was studied at both gene and protein level. Transcripts of *OfHEX3* could be detected in various tissues dissected from the fifth-instar day-3 larvae, including integument, midgut, fat body, Malpighian tubule, trachea, and testis. The highest gene expression was found in the testis (Fig. 3A) while the protein was found mainly in the fat body and testis (Fig. 3B). To further confirm the tissue specificity of OfHex3, we used the larger model insect *B. mori* to allow better tissue separation.

The tissue distribution of Hex3 from *B. mori* agreed well with those from *O. furnacalis*, specifically BmHex3 was mainly found in the fat body and testis but not in the integument, midgut, or ovary of the fifth-instar day-5 larvae (Fig. S5). Additional bands were found in samples isolated from trachea and testis from either *O. furnacalis* or *B. mori,* but only one band was found in samples of molting fluid and fat body. As the Hex3 antibody has been proved very specific, these additional bands could be partially degraded Hex3.

**Figure 4:**
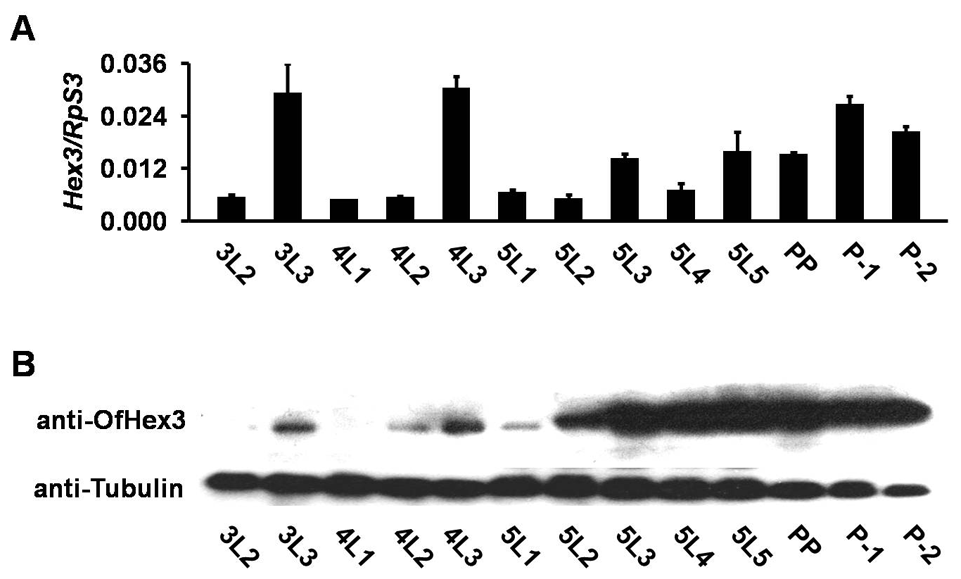
Expression pattern of OfHex3 during development. (**A**) Gene expression level of *OfHEX3* during development of *Ostrinia furnacalis*. Gene expression level was detected from the whole body of *O. furnacalis.* Numbers indicate relative gene expression levels compared with the housekeeping gene *OfRpS3*. (**B**) Protein expression profile of OfHex3 during development of *O. furnacalis*. Total protein was extracted from the whole body of *O. furnacalis* and the protein level of OfHex3 was detected using Hex3 specific antibody. Tubulin was chosen as a loading control. 3L2–5L5: third-instar day-2 to fifth-instar day-5; PP: prepupa; P-1, P-2: pupa day-1 and day-2.

The gene expression levels of *OfHEX3* from day-2 third-instar larvae to day-2 pupae were detected using real-time PCR. The results indicated that *OfHEX3* was up-regulated during larval–larval molting in day-3 third and fourth instars and during larval–pupal molting in the day-1 pupae (Fig. 4A). The protein distribution of OfHex3 in the whole body during development was detected with rabbit anti-Hex3 antibody. As shown in Fig. 4B, the protein level of OfHex3 was up-regulated on the last day of the third and fourth instars, and the highest levels were in day-2 fifth-instar larvae through day-2 pupae (Fig. 4B).

### Subcellular Localization of OfHex3 in Insect Cell Line Sf9

**Figure 5:**
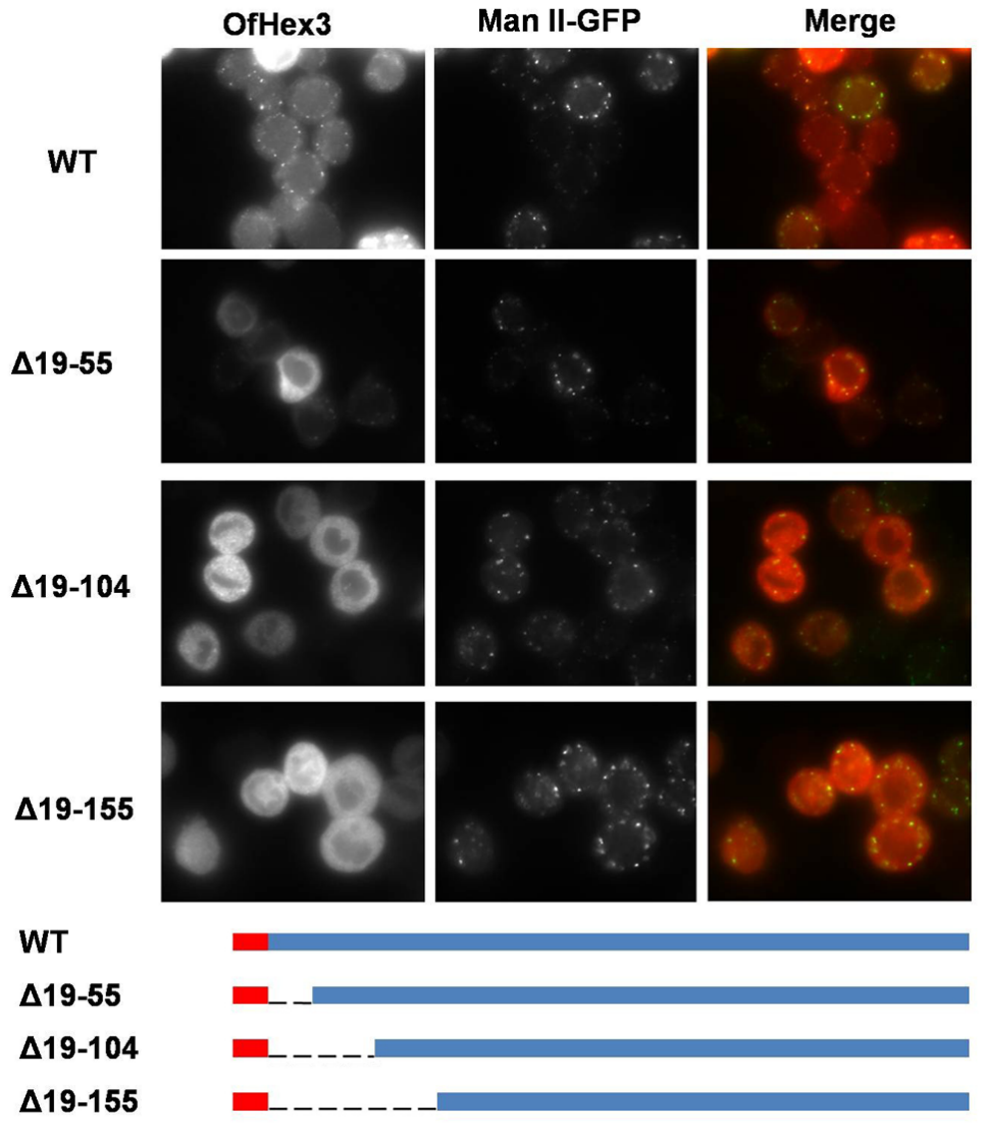
Subcellular localization of OfHex3 in the insect cell line Sf9. OfHex3 was expressed in insect cell line Sf9 fused with N-terminal V5 tag. It was co-transfected with Golgi marker Man II-GFP. The subcellular localizations of the recombinant proteins were detected through immunofluorescence using anti-V5 antibody. The red signal indicates recombinant protein and the green signal indicates Man II-GFP in merge.

The subcellular localizations of OfHex3 were studied using the insect cell line Sf9. Immunofluorescence showed that the recombinant OfHex3 proteins were in puncta structures that co-localized with Golgi marker Man II-GFP, indicating a possible cellular localization of OfHex3 in the Golgi apparatus (Fig. 5).

Several N-terminal truncations (Δ19–55, Δ19–104, and Δ19–155, Fig. 5) of OfHex3 were constructed to investigate whether the subcellular localization was influenced by its N-terminal sequence. For the Δ19–55, although some proteins were in cytoplasm, puncta structures co-localized with Golgi marker Man II-GFP were still found (Fig. 5). The truncations Δ19–104 and Δ19–155 were all in the cytoplasm (Fig. 5), indicating that the N-terminal sequence determined the subcellular localization of OfHex3.

## Discussion

Insect molting fluid contains Hex, which participates in the degradation of old cuticle during molting. Hex is encoded by genes that belong to four groups to function in different life processes. So far, only Hex1 was proved to be present in molting fluid and to function as a chitinolytic enzyme involved in the degradation of cuticle chitin [9], [10], [29]. Here, we showed that OfHex3, the class III Hex from *O. furnacalis*, was also in the molting fluid, too. This is the first report of a class III Hex that functions as a molting fluid enzyme besides playing roles in sperm–egg recognition.

The enzymatic properties of the recombinant OfHex3 revealed its role as a chitinolytic enzyme. First, OfHex3 could efficiently hydrolyze (GlcNAc)_2_ with *k*_cat_ value of 21.07 s^−1^, which was faster than other tested substrates such as *p*NP-β-GlcNAc, *p*NP-β-GalNAc, and GlcNAcβ1,2Man. Second, OfHex3 did not show substrate inhibition at high substrate concentration (10 mM (GlcNAc)_2_), unlike OfHex1, which showed substrate inhibition at 0.2 mM (GlcNAc)_2_ [34]. Also, OfHex3 interacted with OfHex1 in the molting fluid according to the pull-down assay. Therefore, we hypothesized that OfHex3 might cooperate with OfHex1 in cuticle degradation during molting. This speculation was supported by a previous study of Hex3 in *T. castaneum* and *D. melanogaster.* RNAi of *HEX3* (*TcNAG2*) from *T. castaneum* caused lethal phenotypes during larval–larval, larval–pupal and pupal–adult transitions, and the pharate adults could not fully enclose from the pupal cuticle [5]. The enzymatic study of Hex3 (DmHexo2) from *D. melanogaster* showed that it could hydrolyze chitotriose [30].

Notably, OfHex3 was very different from OfHex1 in terms of enzymatic properties. OfHex1 exhibited higher affinity and activity toward chitooligosaccharides and severe substrate inhibition at low substrate concentration (0.2 mM for (GlcNAc)_2_) [34]. In contrast, OfHex3 showed much lower activity for chitooligosaccharides (with a *k*_cat_/*K*_m_ value for (GlcNAc)_2_ 142.8-fold lower than that of OfHex1) but no substrate inhibition. To understand the structural basis for the difference in enzymatic properties, the structure of the catalytic domain of OfHex3 was modeled using that of OfHex1 as template. Striking differences were observed between the +1 subsites of the active pockets of these enzymes. The active pocket of OfHex3 was wider than that of OfHex1 (Fig. 1C). The OfHex1 Trp^490^ that is vital for substrate binding was absent in OfHex3 [10]. The lack of substrate inhibition for OfHex3 might be attributed to the absence of Trp^490^, because the mutation of Trp^490^ to Ala^490^ relieved the substrate inhibition of OfHex1 [34]. We deduced that Hex3 would be an alternative chitinolytic hexosaminidase in molting fluid when substrates concentration tolerance is an issue for Hex1.

In addition to the enzymatic analysis, both the gene transcription and translation levels indicated OfHex3’s involvement in molting. During the development of *O. furnacalis*, both gene and protein levels of OfHex3 were highly up-regulated during larval–larval molting and larval–pupal metamorphosis, suggesting that OfHex3 was involved in chitin degradation during molting.

In summary, OfHex3, a class III Hex, cooperated with OfHex1, a class I Hex, to function as chitinolytic enzymes in the molting fluids of insect.

## Supporting Information

Figure S1 **The cloning strategy of** ***OfHEX3*** **gene.** The full length cDNA of *OfHEX3* was determined by 4 fragments. Fragment 1was the PCR products. Fragment 2 was obtained by 3′-RACE and fragments 3 and 4 were the products of 5′-RACE.
(TIF)

Figure S2 **Multiple sequence alignment of OfHex3 with other insect β-** ***N*** **-acetyl-D-hexosaminidases.** Structure-based multiple sequence alignments of OfHex3 and other insect Hexes were performed with PROMALS3D using the crystal structure of OfHex1 (PDB code: 3NSM) as structure input. Sequence alignment was performed by using the software ESpript 2.2.
(TIF)

Figure S3 **SDS-PAGE and western blot analysis of the recombinant OfHex3.** Proteins were separated by 10% SDS-PAGE. The molecular weight of the recombinant OfHex3 was reduced by 15 kDa after glycopeptidase F treatment. His-tag antibody was used for western blot to detect the recombinant OfHex3.
(TIF)

Figure S4 **Hex3 antibody recognizing BmHex3 from** ***Bombyx mori*** **.** The gene encoding Hex3 (Genbank ID: NM_001085364) from *B. mori* was cloned into the vector pET22b and expressed in *E. coli*. Protein expression was induced by 1 mM of IPTG for 4 hours at 37°C. Both SDS-PAGE and western blot were applied to determine the expression of the recombinant BmHex3 and antibody specificity. Lane 1, 3: cell lysates of *E. coli* harboring the expression vector pET22b-BmHex3; 2, 4: cell lysates of *E. coli* harboring the vector pET22b alone. Arrow indicates the recombinant BmHex3.
(TIF)

Figure S5 **Protein expression profile of Hex3 protein in** ***Bombyx mori*** **.** Proteins were extracted from different tissues of fifth-instar day-5 *B. mori*. The protein expression profile of Hex3 was detected through western blot using Hex3 specific antibody. IN: integument, FB: fat body, MG: midgut, SG: silk gland, MT: Malpighian tubule, TR: trachea, OV: ovary, TE: testis. Tubulin was chosen as a loading control.
(TIF)

Table S1 **Primers used in cloning of** ***OfHEX3.***
(DOCX)

Table S2 **Primers used for Real-Time PCR.**
(DOCX)
