# Supplementary figures and images for: A Sperm–Plasma β-N-Acetyl-D-Hexosaminidase Interacting with a Chitinolytic β-N-Acetyl-D-Hexosaminidase in Insect Molting Fluid
Source: PLoS One. 2013 Aug 12;8(8):e71738. doi: 10.1371/journal.pone.0071738 (PMC3741120; doi:10.1371/journal.pone.0071738)

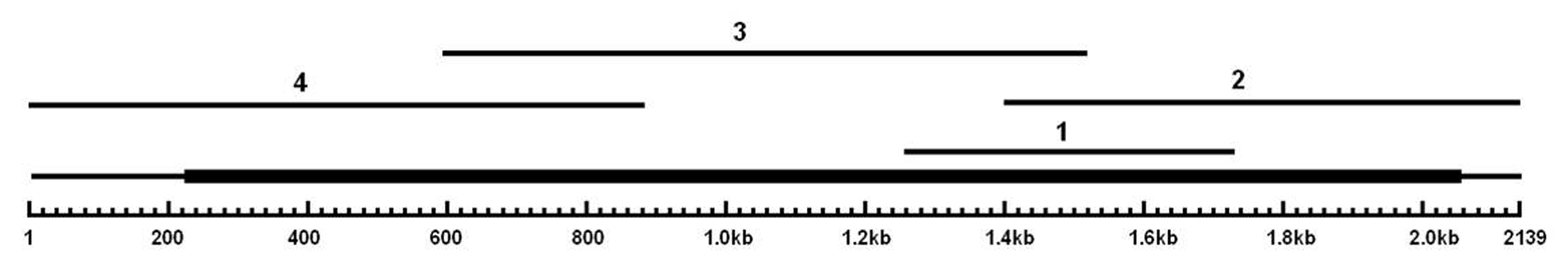

Supplement: Figure S1 — The cloning strategy of OfHEX3 gene. The full length cDNA of OfHEX3 was determined by 4 fragments. Fragment 1was the PCR products. Fragment 2 was obtained by 3′-RACE and fragments 3 and 4 were the products of 5′-RACE. (TIF) [file pone.0071738.s001.tif]

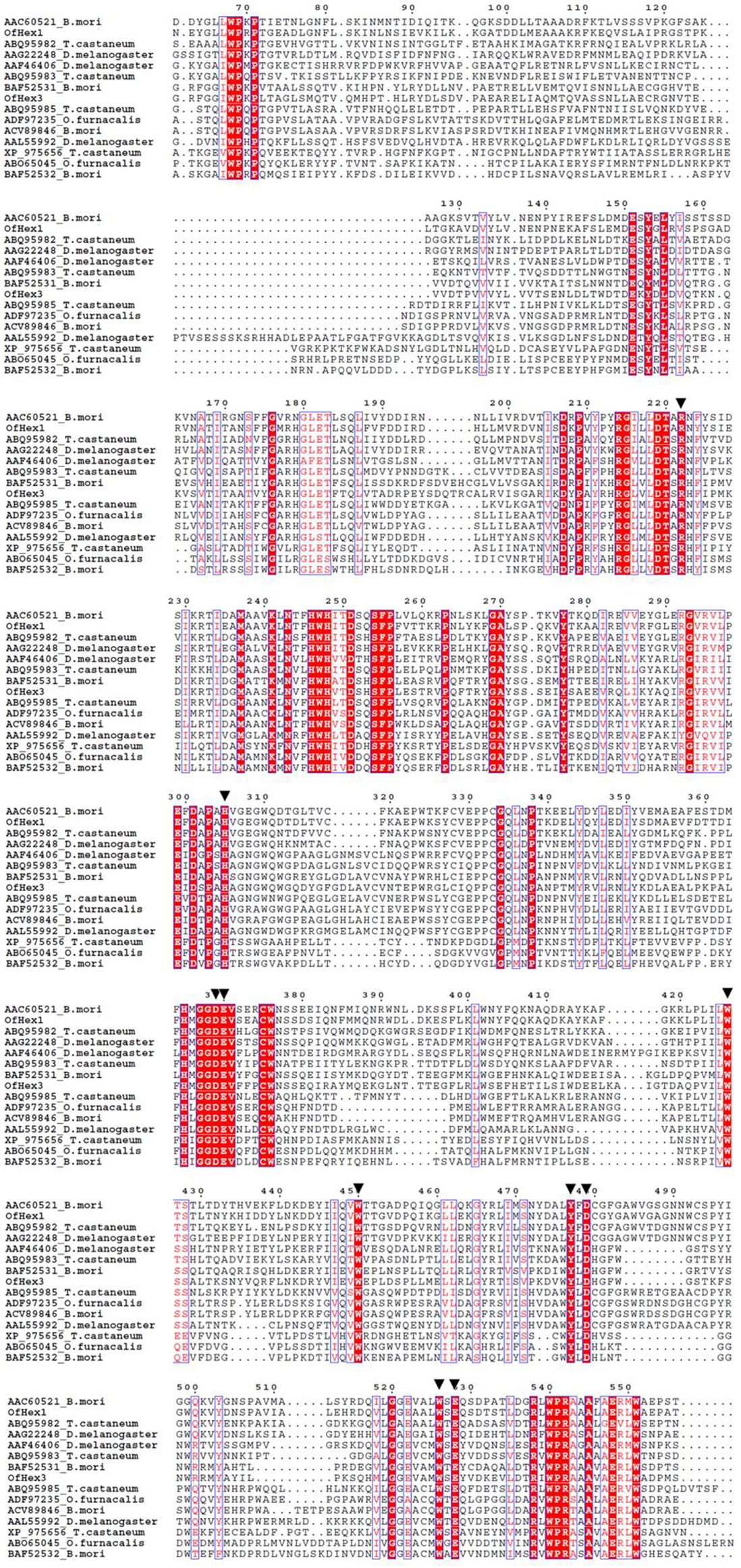

Supplement: Figure S2 — Multiple sequence alignment of OfHex3 with other insect β- N -acetyl-D-hexosaminidases. Structure-based multiple sequence alignments of OfHex3 and other insect Hexes were performed with PROMALS3D using the crystal structure of OfHex1 (PDB code: 3NSM) as structure input. Sequence alignment was performed by using the software ESpript 2.2. (TIF) [file pone.0071738.s002.tif]

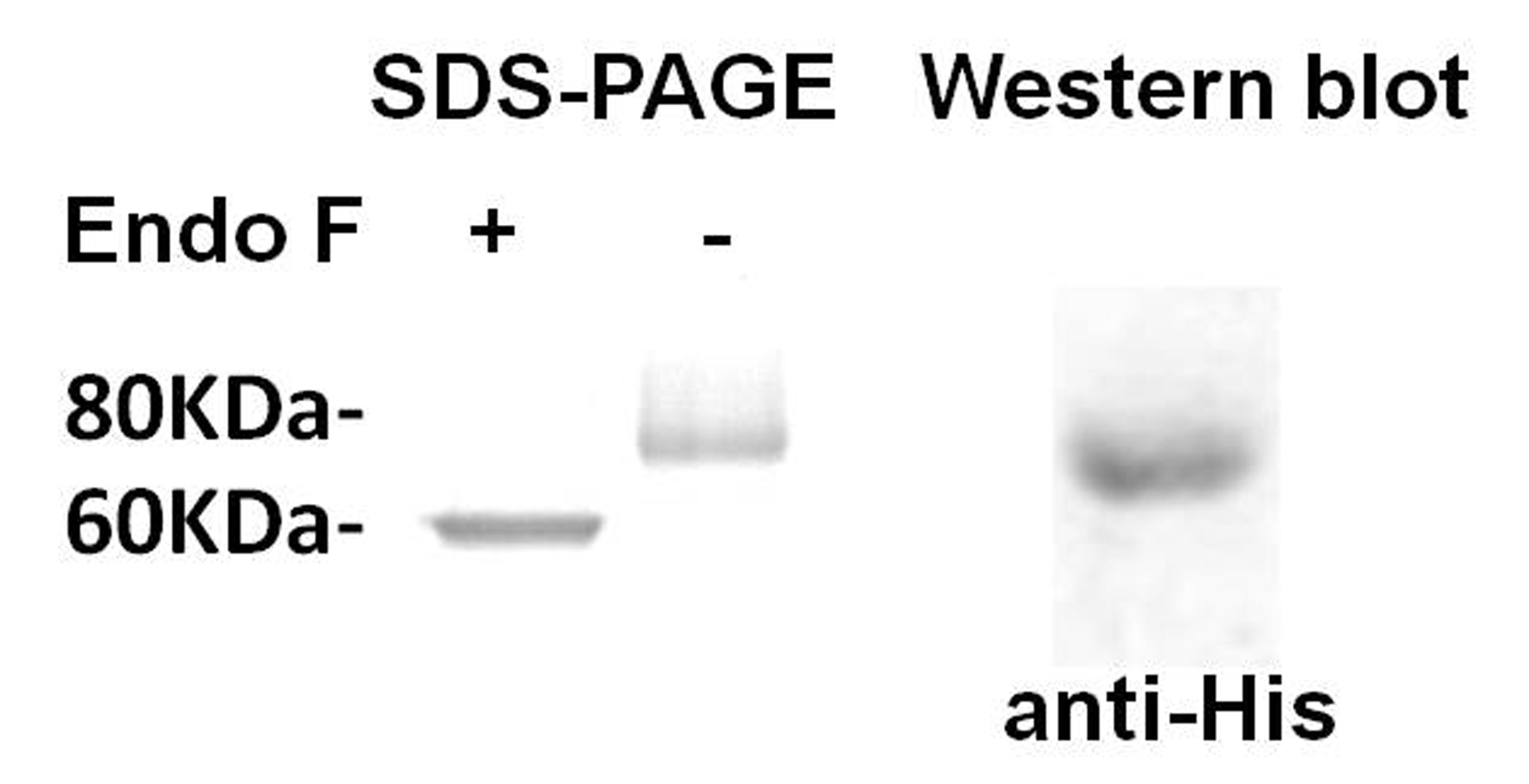

Supplement: Figure S3 — SDS-PAGE and western blot analysis of the recombinant OfHex3. Proteins were separated by 10% SDS-PAGE. The molecular weight of the recombinant OfHex3 was reduced by 15 kDa after glycopeptidase F treatment. His-tag antibody was used for western blot to detect the recombinant OfHex3. (TIF) [file pone.0071738.s003.tif]

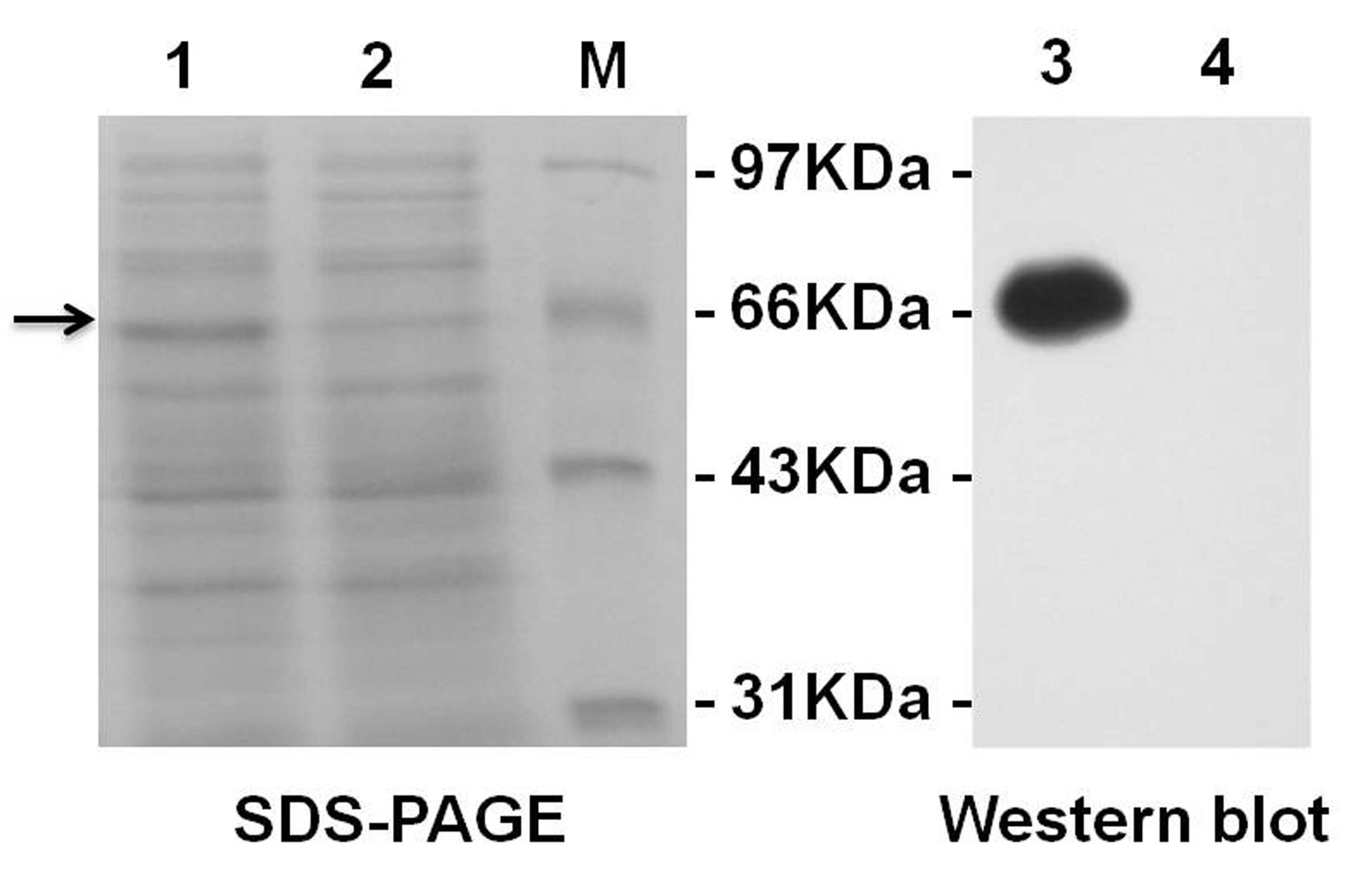

Supplement: Figure S4 — Hex3 antibody recognizing BmHex3 from Bombyx mori . The gene encoding Hex3 (Genbank ID: NM_001085364) from B. mori was cloned into the vector pET22b and expressed in E. coli. Protein expression was induced by 1 mM of IPTG for 4 hours at 37°C. Both SDS-PAGE and western blot were applied to determine the expression of the recombinant BmHex3 and antibody specificity. Lane 1, 3: cell lysates of E. coli harboring the expression vector pET22b-BmHex3; 2, 4: cell lysates of E. coli harboring the vector pET22b alone. Arrow indicates the recombinant BmHex3. (TIF) [file pone.0071738.s004.tif]

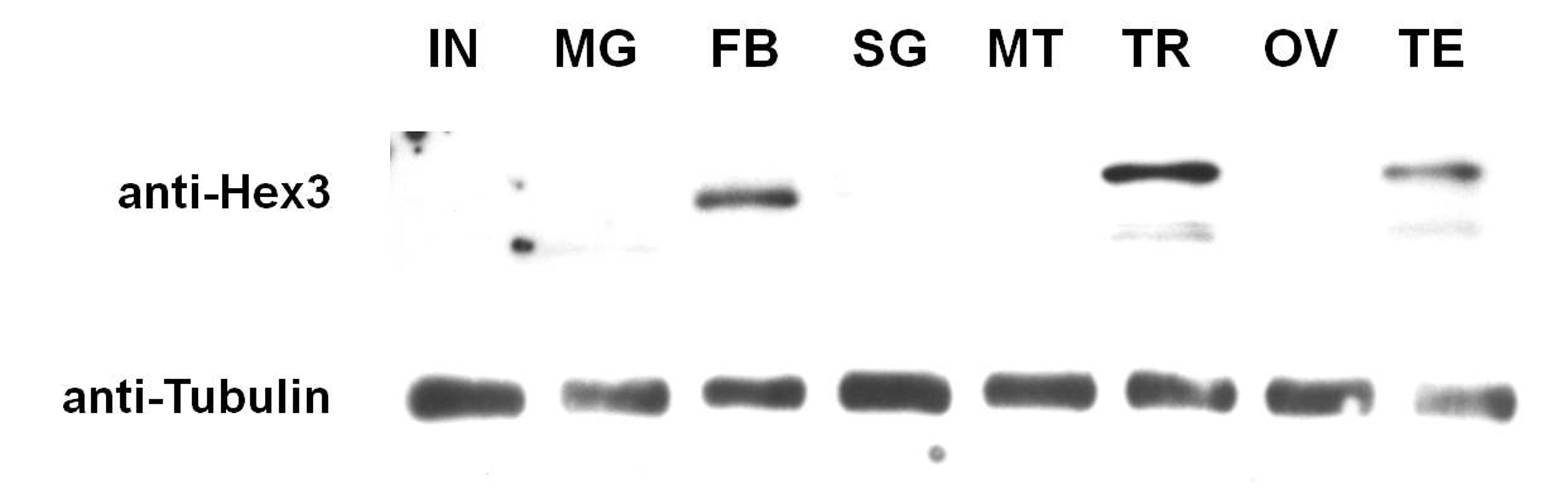

Supplement: Figure S5 — Protein expression profile of Hex3 protein in Bombyx mori . Proteins were extracted from different tissues of fifth-instar day-5 B. mori. The protein expression profile of Hex3 was detected through western blot using Hex3 specific antibody. IN: integument, FB: fat body, MG: midgut, SG: silk gland, MT: Malpighian tubule, TR: trachea, OV: ovary, TE: testis. Tubulin was chosen as a loading control. (TIF) [file pone.0071738.s005.tif]
