# Supplementary material for: A Sperm–Plasma β-N-Acetyl-D-Hexosaminidase Interacting with a Chitinolytic β-N-Acetyl-D-Hexosaminidase in Insect Molting Fluid
Source: PLoS One. 2013 Aug 12;8(8):e71738. doi: 10.1371/journal.pone.0071738 (PMC3741120; doi:10.1371/journal.pone.0071738)
Supplement: Table S1 — Primers used in cloning of OfHEX3. (DOCX) [file pone.0071738.s006.docx]

**Table S1. Primers used in cloning of *OfHEX3***

| Fragment. | Size(bp) | Direction | Sequence(5′-3′) | Aminal acids |
| --- | --- | --- | --- | --- |
| 1 | 475 | F1 | TGYATNCARCCNCCNTGYGG | CIQPPCG |
|  |  | R1 | AANCCRTGRTCNARRTACCA | WYLDHGF |
| 2 | 728 | 3F1 | TGGGTGGTGATGAGGTATTCTTC | MGGDEVFF |
|  |  | 3F2 | AAGCGAGCAAATCCGAGCATACA | SEQIRAY |
|  |  | 3' race outer | TACCGTCGTTCCACTAGTGATTT | / |
|  |  | 3' race inner | CGCGGATCCTCCACTAGTGATTTCACTAT | / |
| 3 | 722 | 5' race outer | CATGGCTACATGCTGACAGCCTA | / |
|  |  | 5' race inner | CGCGGATCCACAGCCTACTGATGATCAGTCGATG | / |
|  |  | 5R1 | AGCCGAGACGAAGCAGTT | ELLRLG |
|  |  | 5R2 | TCGGTCCCTATTGCCTTC | KAIGTD |
| 4 | 888 | 5' race outer | CATGGCTACATGCTGACAGCCTA | / |
|  |  | 5' race inner | CGCGGATCCACAGCCTACTGATGATCAGTCGATG | / |
|  |  | 5R3 | CCGTATCTTGTAAACTGTGGC | PQFTRYG |
|  |  | 5R4 | GCAGGGTAGTCCTTGATTCTTG | ARIKDYPA |
