# Supplementary material for: A Sperm–Plasma β-N-Acetyl-D-Hexosaminidase Interacting with a Chitinolytic β-N-Acetyl-D-Hexosaminidase in Insect Molting Fluid
Source: PLoS One. 2013 Aug 12;8(8):e71738. doi: 10.1371/journal.pone.0071738 (PMC3741120; doi:10.1371/journal.pone.0071738)
Supplement: Table S2 — Primers used for Real-Time PCR. (DOCX) [file pone.0071738.s007.docx]

**Table S2. Primers used for Real-Time PCR.**

| Gene | F-primer(5′-3′) | R-primer(5′-3′) | Size(bp) |
| --- | --- | --- | --- |
| *OfHex3* | GAGGAGCTGAAGGCAATAGG | GGACTGTCTAAGGGTTCCCA | 137 |
| *OfRpS3* | TGCAACGACTACGTCAACACC | TCGGGCTGCGGTTTCTT | 128 |
